# Declining Comorbidity Burden Among Patients During First Hospitalization Associated with Thyroid Cancer in Poland: A Nationwide Study, 2012–2024

**DOI:** 10.3390/jcm15218481

**Published:** 2026-10-31

**Authors:** Dorota Szydlarska, Katarzyna Lewtak, Marta Zuzanna Ciechomska, Anna Poznańska, Andrzej Śliwczyński, Krzysztof Kanecki

**Affiliations:** 1 Department of Digital Medicine, Implementation and Innovation, National Medical Institute of the Ministry of the Interior and Administration, Woloska 137, 02-507 Warsaw, Poland; 2 Department of Family Medicine, Medical University of Warsaw, 02-097 Warsaw, Poland; 3 Department of Social Medicine and Public Health, Medical University of Warsaw, 02-091 Warsaw, Poland; 4 National Medical Institute of the Ministry of the Interior and Administration, Woloska 137, 02-507 Warsaw, Poland; 5 Department of Population Health Monitoring and Analysis, National Institute of Public Health NIH–National Research Institute, 00-791 Warsaw, Poland; 6 Institute of Psychology and Human Sciences, WSEI University, Lubelska Akademia WSEI, 20-209 Lublin, Poland

**Keywords:** thyroid cancer, comorbidity, cardiovascular disease, endocrine disorders, epidemiology, nationwide study

## Abstract

**Background:** Thyroid cancer (TC) is the most common endocrine malignancy worldwide. Although the burden of thyroid-related disorders accompanying TC has been extensively studied, less is known about the overall pattern of multimorbidity present at the time of diagnosis. The aim of this study was to characterize hospital-recorded comorbidities among patients during their first hospitalization associated with thyroid cancer in Poland and to evaluate temporal changes in these patterns between 2012 and 2024. **Methods:** This nationwide retrospective cohort study included 60,604 patients with a first thyroid-cancer-associated hospitalization recorded in Poland between 2012 and 2024 using data from the Nationwide General Hospital Morbidity Study. The study included all first hospitalizations with ICD-10 code C73 recorded in the database during the study period. Diagnoses accompanying thyroid cancer were classified according to ICD-10 chapters and analyzed descriptively. For the purposes of this study, incident cases were operationally defined as patients with a first hospitalization associated with ICD-10 code C73 recorded in the database during the study period. Temporal trends in age and major comorbidity groups were assessed using linear regression models. **Results:** A total of 60,604 patients with a first recorded hospitalization associated with TC were identified, including 49,794 women (82.2%) and 10,810 men (17.8%). The mean age at diagnosis was 51.1 ± 15.9 years. Endocrine, nutritional, and metabolic disorders constituted the most common comorbidity group, affecting 20,792 patients (34.3%), followed by cardiovascular diseases (13.6%) and neoplasms other than TC (5.0%). Hypothyroidism was the most frequent endocrine disorder, occurring in 11.5% of patients. During the study period, the mean age at diagnosis decreased from 53.7 years to 49.8 years (*p* < 0.001). A marked temporal decline in the prevalence of recorded comorbidities was observed during the study period, with endocrine/metabolic disorders decreasing from 52.0% to 25.3% and cardiovascular diseases from 19.9% to 7.7% (both *p* < 0.001). Additional malignancies most commonly involved the breast, lung, kidney and ovary. **Conclusions:** Patients with first thyroid-cancer-associated hospitalization recorded in the database frequently present with concomitant endocrine, cardiovascular, and oncological disorders. Over the study period, a substantial decline in the prevalence of recorded comorbidities was accompanied by a reduction in age at diagnosis, suggesting changes in the characteristics of patients recorded during thyroid-cancer-associated hospitalizations over time. These findings may have implications for healthcare planning and survivorship care by highlighting the evolving clinical profile of patients undergoing thyroid-cancer-associated hospitalization.

## 1. Introduction

Thyroid cancer (TC) is currently recognized as the seventh most common malignancy worldwide, while its associated mortality remains relatively low compared with most other cancers [1]. The epidemiology of TC is characterized by a pronounced sex disparity, with incidence rates approximately threefold higher in women than in men [2]. This difference is largely attributable to the predominance of less aggressive histological subtypes among female patients, whereas the incidence of more aggressive variants appears comparable between sexes. Unlike most adult malignancies, TC is frequently diagnosed at a relatively young age, with the mean age at diagnosis reported to be approximately 51 years [3]. Clinicopathological characteristics also vary according to age, as younger individuals are more likely to present with adverse pathological features, including lymph node metastases, capsular invasion, and neurovascular involvement [4].

Significant racial and ethnic differences in TC incidence have also been documented. The highest incidence rates have been reported among White populations, followed by Asian/Pacific Islanders and American Indian/Alaska Native populations, whereas the lowest rates are observed among Black individuals. Furthermore, both male and female individuals of non-Hispanic origin exhibit higher incidence rates than their Hispanic counterparts [5]. Over recent decades, the global burden of TC has increased substantially. Between 1992 and 2018, the incidence of TC rose by more than 200%, while mortality remained relatively stable [6]. Similar trends have been reported worldwide, with the greatest increases observed in younger age groups [7]. According to GLOBOCAN estimates, TC-related mortality remains below 1 per 100,000 population in most countries regardless of sex [8].

The growing incidence of TC, combined with advances in surgical management, radioactive iodine therapy, and long-term follow-up strategies, has led to a steadily expanding population of TC survivors. Consequently, the focus of clinical care has broadened beyond cancer-specific outcomes to include the long-term health status of affected individuals [9]. Increasing evidence suggests that concomitant chronic diseases frequently influence the clinical course of patients with TC and may play a greater role in long-term prognosis than the malignancy itself [10,11]. Metabolic disorders such as obesity, diabetes, and dyslipidemia are commonly observed in this population and may affect both tumor characteristics and therapeutic decision-making [12,13].

Despite the generally favorable prognosis of TC, important knowledge gaps remain regarding the burden and clinical implications of comorbidities. Previous studies have suggested that patients with TC may experience an increased risk of cardiovascular disease, second primary malignancies, and other chronic conditions [14]. However, the available evidence remains inconsistent. While some reports indicate a higher risk of non-cancer morbidity and mortality among TC survivors, others suggest that long-term outcomes may be comparable to those observed in the general population [15,16]. Furthermore, the impact of metabolic disorders and multimorbidity on treatment intensity, perioperative risk, and long-term survival remains incompletely understood [12,17]. Although previous studies have evaluated selected comorbidities, including cardiovascular disease, metabolic disorders, and second primary malignancies, most focused on individual conditions or their impact on prognosis. Nationwide population-based data characterizing the overall spectrum of comorbidities accompanying thyroid cancer at the time of diagnosis remain limited. Furthermore, little is known about how these comorbidity patterns have changed over time. Addressing this knowledge gap may improve understanding of the evolving clinical profile of patients with thyroid cancer and inform future healthcare planning.

The concept of multimorbidity is particularly relevant in TC because patient outcomes are influenced not only by tumor biology but also by competing health conditions and treatment-related consequences. The prevalence of recorded comorbidities, treatment-related late effects, and competing mortality risks represent interconnected factors that collectively shape prognosis and healthcare utilization [16]. In many patients with differentiated TC, non-thyroidal causes of death may ultimately exceed thyroid cancer-specific mortality, emphasizing the importance of a holistic approach to patient care [15,18,19].

Therefore, the aim of the present study was to characterize the spectrum of coexisting disorders among patients with first thyroid-cancer-associated hospitalization recorded in the database in Poland and to identify the most common non-endocrine conditions accompanying first-time hospitalizations associated with thyroid cancer in a nationwide population-based cohort.

## 2. Materials and Methods

### 2.1. Data Source and Study Design

This retrospective nationwide cohort study was based on data obtained from the Nationwide General Hospital Morbidity Study maintained by the National Institute of Public Health NIH–National Research Institute. The database contains anonymized hospital-discharge records reported by Polish hospitals and provides nationwide coverage of inpatient care.

The database contains anonymized information on all hospital discharges reported by Polish hospitals. Patients were eligible for inclusion if they were hospitalized between 1 January 2012 and 31 December 2024 and had a diagnosis of TC (ICD-10 code C73) recorded either as the principal diagnosis or among secondary discharge diagnoses. To estimate incident TC cases, only the first hospitalization associated with ICD-10 code C73 during the study period was included for everyone. Repeated hospitalizations of the same patient were excluded to avoid duplicate counting.

The final study cohort consisted of patients with a first thyroid-cancer-associated hospitalization recorded in the database during the observation period.

The study protocol, including the retrospective analysis of anonymized hospital-discharge records, was approved by the Ethics Committee of the Medical University of Warsaw (AKBE/220/2026).

### 2.2. Study Population

The study population comprised all patients with first thyroid-cancer-associated hospitalization recorded in the Nationwide General Hospital Morbidity Study database between 2012 and 2024. Comorbidities were defined as diagnoses recorded during the analyzed hospitalization and do not represent the complete lifetime morbidity profile of the patient. All diagnoses recorded in hospital discharge records were considered and subsequently classified according to major ICD-10 categories.

Demographic characteristics including age, sex, calendar year of hospitalization, and coexisting diagnoses recorded at hospital discharge were extracted and analyzed. Information on histological subtype, tumor stage, treatment modality, and long-term outcomes was not available in the source database.

### 2.3. Statistical Analysis

Continuous variables were summarized as means and standard deviations (SDs) or medians. Categorical variables were presented as absolute numbers and percentages. Diagnoses were classified according to major ICD-10 categories. Sex-specific descriptive analyses were performed for demographic characteristics; however, temporal trend analyses were conducted for the overall study population.

Temporal changes in mean patient age and the annual prevalence (%) of major comorbidity groups were evaluated using simple linear regression models, with calendar year treated as the independent variable and annual values of each analyzed parameter as dependent variables.

Results were considered statistically significant at a two-sided *p*-value < 0.05. All statistical analyses were performed using Statistica version 13.3 (TIBCO Software Inc., Palo Alto, CA, USA).

## 3. Results

A total of 60,604 first-time hospitalizations associated with TC were included in the analysis. Women accounted for 49,794 cases (82.2%), whereas men represented 10,810 cases (17.8%) (Table 1). The mean age of the study population was 51.1 ± 15.9 years. Patients were significantly younger in recent years compared with the beginning of the study period.

Among the 60,604 first-time hospitalizations associated with TC, a wide spectrum of coexisting endocrine disorders was identified (Table 2). The most frequently recorded conditions included hypothyroidism (E03), non-toxic goiter (E04), hyperthyroidism (E05), and thyroiditis (E06). In addition, several other endocrine disorders were observed, including hyperparathyroidism (E21), pituitary disorders (E22), benign adrenal neoplasms (D35.0), and benign pituitary neoplasms (D35.2). These findings indicate that patients recorded in the database frequently present with concomitant endocrine abnormalities, reflecting the complexity of endocrine pathology in this population.

Among patients hospitalized, a broad spectrum of non-endocrine diagnoses was identified (Table 3). The most common comorbidity groups were cardiovascular diseases (13.6%) and neoplastic disorders (5.0%). Although endocrine disorders predominated, cardiovascular diseases represented the most frequent non-endocrine comorbidity group. More than one in eight patients had at least one cardiovascular diagnosis recorded during hospitalization.

Significant temporal changes were observed throughout the study period. The mean age at diagnosis decreased significantly from 53.7 years in 2012 to 49.8 years in 2024, corresponding to an average reduction of approximately 0.4 years per calendar year (*p* < 0.001) (Table 4). Women were diagnosed at a younger age than men (mean age, 50.7 vs. 53.2 years).

Overall, this represents a decline of nearly four years in the average age at diagnosis over the 13-year observation period. The strong coefficient of determination indicates that calendar year explained a substantial proportion of the variability in patient age, suggesting a consistent and progressive trend rather than random fluctuations. Endocrine, nutritional and metabolic disorders demonstrated a marked decreasing trend during the study period. Their prevalence declined from 52.0% in 2012 to 25.3% in 2024, corresponding to an average reduction of 1.94 percentage points per year. The observed trend showed a strong linear association with calendar year (*p* < 0.001) (Table 4). Cardiovascular comorbidities demonstrated a significant decreasing trend during the study period, declining from 19.9% in 2012 to 7.7% in 2024. The average reduction was 0.95 percentage points per year, with a strong linear association between calendar year and comorbidity prevalence (*p* < 0.001) (Table 4).

**Figure 1:**
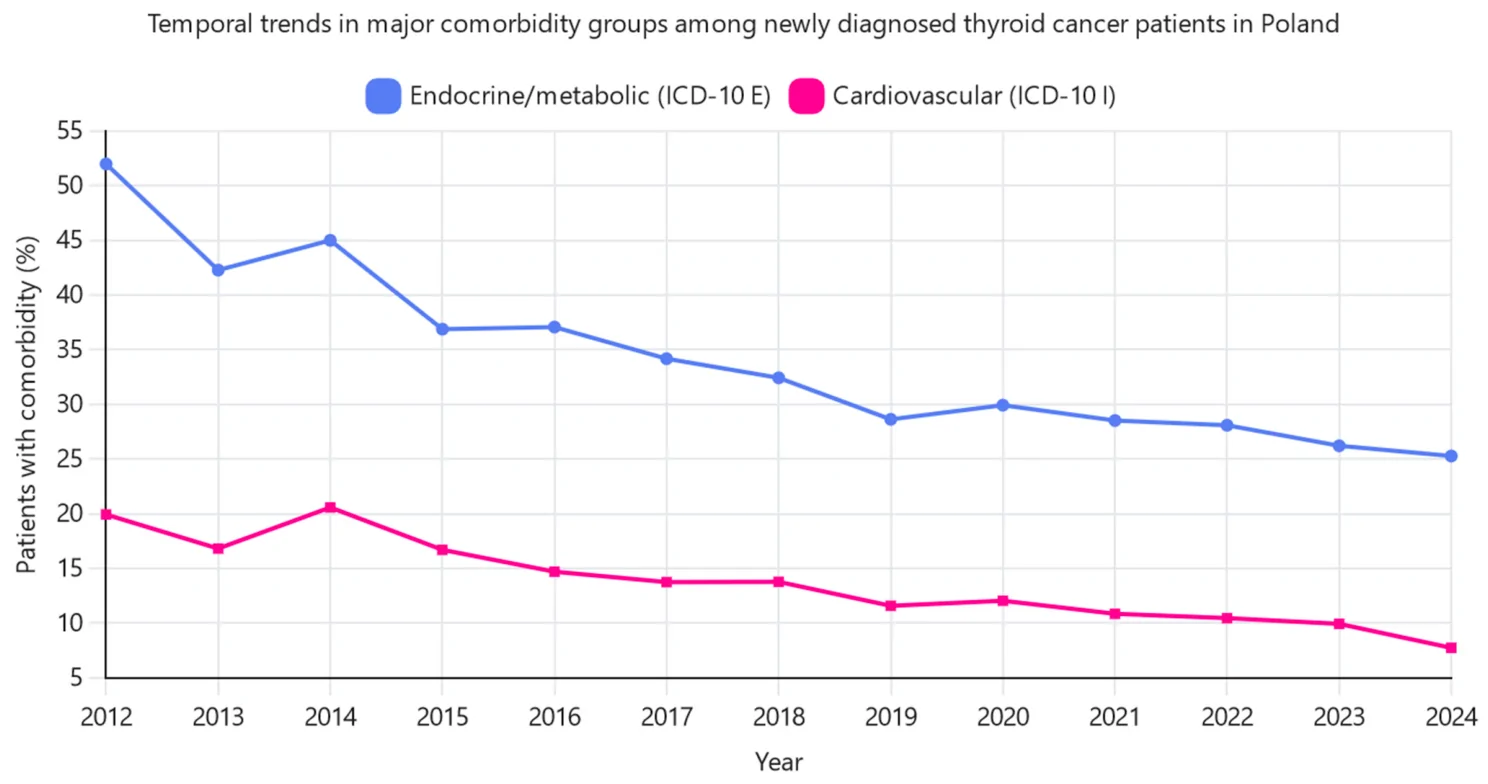
Temporal changes in the yearly prevalence (%) of endocrine/metabolic (ICD-10 chapter E) and cardiovascular (ICD-10 chapter I) comorbidities among patients with first thyroid-cancer-associated hospitalization recorded in the database in Poland between 2012 and 2024. Lines represent annual prevalence estimates.

Figure 1 illustrates the temporal trends in the two most prevalent comorbidity categories. Both endocrine/metabolic and cardiovascular disorders demonstrated a progressive decline throughout the study period, with the most pronounced reduction observed after the middle of the observation period.

## 4. Discussion

The present nationwide study demonstrated a progressive decline in patient age and hospital-recorded diagnoses among individuals with first thyroid-cancer-associated hospitalization recorded in the database in Poland between 2012 and 2024. To our knowledge, few population-based studies have examined the spectrum of non-endocrine conditions accompanying TC. Previous studies have demonstrated substantial heterogeneity in the reported prevalence of comorbidities among patients with TC, with estimates ranging from approximately 2% to more than 70%, depending on study design, patient characteristics, and the definitions used for multimorbidity [10,20]. Older age has consistently been identified as one of the strongest determinants of the prevalence of recorded comorbidities in this population [10,20].

An important observation was the relatively young age of the study population and the progressive decrease in mean age throughout the study period. Between 2012 and 2024, the average age at diagnosis declined by almost four years. This trend may reflect increasing use of thyroid ultrasonography, fine-needle aspiration biopsy, and cross-sectional imaging performed for unrelated clinical indications. As diagnostic procedures become more widely available, thyroid nodules are detected more frequently, resulting in increased identification of TC at earlier stages and in younger individuals. The magnitude of this change is noteworthy. A reduction of nearly four years in the mean age of patients recorded during first thyroid cancer-related hospitalization over a relatively short period suggests a substantial shift in the clinical profile of this population. Importantly, the decrease in age occurred simultaneously with a marked decline in the prevalence of endocrine and cardiovascular comorbidities, indicating that temporal changes were not limited to demographic characteristics alone. This parallel trend supports the hypothesis that patients recorded in more recent years differ substantially from those observed at the beginning of the study period.

The coexistence of endocrine disorders provides additional insight into evolving diagnostic pathways. Hypothyroidism was the most frequently reported thyroid-related condition, occurring in more than one-tenth of patients. The interpretation of hypothyroidism in this population requires caution. Because the database does not contain information on the temporal relationship between thyroid cancer diagnosis, thyroidectomy, and initiation of thyroid hormone replacement therapy, recorded cases of hypothyroidism may represent either pre-existing thyroid dysfunction or postoperative hypothyroidism associated with treatment. These two conditions cannot be differentiated within the available dataset.

However, the proportion of patients with concomitant hypothyroidism appeared to decline over time, whereas diagnoses of non-toxic goiter became relatively more common. This pattern may indicate a shift from thyroid dysfunction-driven investigations toward imaging-driven detection of structural thyroid abnormalities. The increasing number of neck ultrasounds, fine-needle aspiration procedures, and other imaging examinations performed during recent years has likely contributed to more frequent detection of TC among patients with nodular thyroid disease in the absence of overt hormonal dysfunction.

One of the most intriguing findings of the present study was the substantial temporal decline in the prevalence of endocrine and cardiovascular comorbidities. The proportion of endocrine and metabolic disorders decreased from 52.0% in 2012 to 25.3% in 2024, while cardiovascular diseases decreased from 19.9% to 7.7% during the same period. This trend occurred in parallel with a reduction in the mean age of patients diagnosed with TC, from 53.7 years to 49.8 years. Notably, the decline in endocrine and metabolic disorders was proportionally greater than the decline observed for cardiovascular diseases. Given the close relationship between TC and other thyroid disorders, this finding may indicate substantial changes in the pathways leading to TC detection and hospitalization over time. The simultaneous decline in age and endocrine recorded comorbidities suggests that patients recorded in more recent years may represent a clinically different population than those hospitalized at the beginning of the study period.

The observed decline in the prevalence of recorded comorbidities should be interpreted with caution. The observed decline in the prevalence of recorded comorbidities should not be interpreted solely as evidence of improving health status among patients with thyroid cancer. Alternative explanations should also be considered. Increasing use of outpatient diagnostic pathways, day-surgery management, and evolving hospital admission criteria may have altered the spectrum of patients requiring inpatient care. Consequently, part of the observed temporal changes may reflect healthcare system factors and documentation practices rather than true epidemiological shifts in the burden of accompanying diseases. Furthermore, because the database does not contain information on disease stage, histological subtype, treatment modality, or outpatient diagnoses, it is not possible to disentangle the relative contributions of changes in patient characteristics, diagnostic pathways, healthcare delivery, and coding practices to the observed trends.

Several non-mutually exclusive explanations may account for this trend. First, increasing use of thyroid ultrasonography and other imaging modalities may have resulted in earlier detection of TC among younger individuals with fewer chronic conditions. Second, temporal changes in hospital admission practices and diagnostic pathways may have altered the clinical profile of patients recorded in the database. Third, the decline in comorbidity prevalence was accompanied by a decrease in mean age at hospitalization, suggesting that demographic changes may partly explain the observed pattern. Additional healthcare-system factors should also be considered. During the study period, increasing use of outpatient management, development of day-surgery pathways, and evolving hospital admission criteria may have altered the spectrum of patients requiring inpatient care. Consequently, part of the observed decline in recorded comorbidities may reflect changes in healthcare delivery rather than true changes in population health.

Fourth, changes in coding practices and reporting of secondary diagnoses over time cannot be excluded. The COVID-19 pandemic may have contributed to the observed temporal patterns. During 2020–2021, substantial disruptions in healthcare delivery, postponement of elective procedures, and changes in hospital admission practices occurred worldwide. Consequently, part of the variation observed during this period may reflect pandemic-related healthcare system effects rather than true epidemiological changes.

Furthermore, temporal changes in hospital reimbursement and administrative reporting systems may have influenced the recording of secondary diagnoses. Consequently, part of the observed decline in comorbidity prevalence may reflect evolving documentation practices rather than true changes in the burden of coexisting diseases. In addition, age is one of the strongest determinants of multimorbidity. Therefore, the nearly four-year reduction in mean age observed during the study period may have contributed independently to the decreasing prevalence of endocrine and cardiovascular comorbidities. Because the present database does not allow formal age standardization of hospitalization records, the relative contributions of demographic changes and changes in the underlying population cannot be fully disentangled.

Finally, because the database was available only from 2012 onward, some patients classified as first hospitalizations may have had TC diagnosed before the beginning of the observation period. Consequently, a portion of the observed temporal trend may reflect methodological factors related to case ascertainment rather than true changes in the underlying burden of comorbidity.

A major finding of the present study was the presence of cardiovascular disorders among patients hospitalized with TC. The relatively low frequency of cardiovascular comorbidities should also be interpreted in the context of the demographic characteristics of TC patients. Unlike many other malignancies, TC is frequently diagnosed in middle-aged adults, resulting in a younger study population and, consequently, a lower expected prevalence of age-related chronic conditions. Therefore, although cardiovascular diseases represented one of the most common groups of non-endocrine diagnoses in our cohort, their absolute frequency was lower than would be anticipated in malignancies predominantly affecting elderly patients. Cardiovascular disease has repeatedly been identified as the leading non-cancer cause of death among long-term survivors of TC [11,18,21]. Changes in thyroid cancer management during the study period may also have contributed to the evolving clinical profile of hospitalized patients. Contemporary management increasingly relies on risk-adapted approaches, including reduced surgical extent in selected patients and more restrictive use of radioactive iodine therapy. Although treatment-related information was not available in the present database, these changes may have influenced patterns of hospitalization and the prevalence of accompanying comorbidities observed over time.

Several studies have demonstrated that, particularly among patients with differentiated TC and favorable oncological prognosis, the risk of death from cardiovascular disease and other non-thyroidal causes may exceed the risk of death attributable to TC itself [11,15,18,19].

Our findings should also be interpreted in the broader context of metabolic health. Previous studies have identified obesity, diabetes mellitus, dyslipidemia, and other cardiometabolic disorders as among the most prevalent comorbidities in patients with TC [12,13,22,23,24]. Although the current database was not specifically designed to assess the burden of metabolic disease, the coexistence of cardiovascular conditions observed in our study may partly reflect the underlying influence of shared metabolic risk factors. The growing prevalence of obesity and metabolic syndrome worldwide may therefore contribute not only to the burden of chronic disease but also to the overall health profile of patients diagnosed with thyroid malignancies.

The clinical relevance of multimorbidity extends beyond diagnostic complexity. A growing body of evidence indicates that overall survival among patients with TC is strongly influenced by coexisting chronic diseases and competing causes of mortality [11,12,19,25]. Patients with a high prevalence of recorded comorbid conditions or multiple primary malignancies consistently experience worse overall survival than patients affected by thyroid cancer alone [7,20,25].

Respiratory disorders constituted another important group of accompanying diagnoses. Dyspnea, respiratory failure, and pleural effusions were frequently observed, often together with pulmonary metastatic disease. These findings suggest that at least some respiratory diagnoses recorded during hospitalization may represent manifestations of advanced TC rather than independent chronic conditions. Pulmonary metastases constitute one of the most common sites of distant spread in differentiated TC, and respiratory symptoms may therefore reflect disease progression at the time of first hospitalization. The presence of additional malignant neoplasms was also noteworthy. Breast, lung, kidney, and ovarian cancers were the most frequently reported non-thyroid malignancies in our cohort. The presence of additional malignancies in one out of every twenty patients is clinically relevant. Importantly, this proportion reflects malignant neoplasms recorded concurrently during the analyzed hospitalization and should not be interpreted as the lifetime cumulative risk of second primary malignancies among patients with thyroid cancer.

Although the database does not allow assessment of the temporal sequence between cancers, the finding suggests that coexistence of TC with other neoplasms is not uncommon at the time of hospitalization and should be taken into account when interpreting treatment decisions and healthcare utilization. This finding is consistent with previous studies reporting an increased occurrence of second primary malignancies among patients with thyroid cancer [20,23]. Potential explanations include shared genetic susceptibility, common environmental exposures, hormonal influences, and increased medical surveillance after cancer diagnosis. Although causal relationships cannot be established using the present dataset, the coexistence of multiple malignancies highlights the importance of comprehensive oncological assessment in patients with TC. Renal disorders and anemia were relatively uncommon but clinically relevant findings. Both may reflect an increased chronic disease burden, frailty, or advanced malignancy. Although identified in only a small proportion of patients, their presence may influence perioperative risk and overall clinical management [17,21,22,26].

The major strength of this study is its nationwide design and inclusion of all first hospitalizations with a reported diagnosis of TC recorded in Poland over a thirteen-year period. The large sample size minimizes selection bias and provides a unique opportunity to characterize patterns of multimorbidity at the population level. Nevertheless, several limitations should be acknowledged. First, the analysis was based on administrative hospital discharge data and therefore depended on the accuracy of ICD-10 coding. Second, information regarding histological subtype, tumor stage, treatment modalities, and disease outcomes was unavailable. Third, diagnoses recorded during hospitalization may represent pre-existing comorbidities, complications of thyroid cancer, or conditions identified during diagnostic work-up, and these entities cannot be completely distinguished within the available dataset. Finally, the observational design does not allow causal inference.

Taken together, our findings and currently available evidence suggest that the long-term prognosis of patients with TC is determined not only by tumor biology but also by the burden of concomitant diseases and competing mortality risks [11,18,19]. As survival after TC continues to improve, multimorbidity is likely to become an increasingly important determinant of treatment decisions, healthcare utilization, and long-term outcomes. Therefore, TC should be viewed not merely as an isolated endocrine malignancy but as a disease frequently occurring within a broader context of chronic multimorbidity requiring comprehensive and multidisciplinary care.

The observed shift toward younger patients with lower recorded comorbidities may have important implications for healthcare planning. As TC is increasingly diagnosed in healthier individuals, long-term survivorship care may become more relevant than the management of acute cancer-related complications. These findings further support the need for risk-adapted diagnostic approaches to avoid unnecessary investigations and potential overdiagnosis of indolent thyroid tumors. Taken together, the simultaneous decline in age, endocrine disorders, and cardiovascular diseases suggests a substantial evolution in the characteristics of patients recorded during thyroid cancer-related hospitalizations in Poland. The consistency of these trends across major diagnostic categories argues against random variation and indicates a broader change in the population captured by the healthcare system over time.

## 5. Limitations

This study has several limitations. First, the analysis was based on administrative hospital discharge data and therefore relied on the accuracy and completeness of ICD-10 coding. Second, the database did not contain information on histological subtype, TNM stage, molecular characteristics, treatment modalities, or long-term outcomes. Because the source database is anonymized and does not contain patient identifiers enabling record linkage, validation against cancer registry data and linkage with external clinical databases were not feasible.

Consequently, it was not possible to evaluate whether the observed comorbidity patterns differed according to tumor aggressiveness, disease stage, histological subtype, or treatment strategy. Therefore, part of the observed temporal changes may reflect shifts in the composition of thyroid cancer cases captured by the healthcare system rather than true changes in the prevalence of accompanying conditions.

Third, because only hospital-recorded diagnoses were available, recorded conditions may represent pre-existing comorbidities, complications of thyroid cancer, or disorders identified during diagnostic work-up. Furthermore, the reported diagnoses reflect conditions recorded during the analyzed hospitalization and should not be interpreted as a comprehensive assessment of the lifetime prevalence of recorded comorbidities. Finally, because information before 2012 was unavailable, some patients classified as first hospitalizations may have been diagnosed with TC before the beginning of the study period. Accordingly, part of the observed decline in age and comorbidity burden may reflect methodological factors related to case ascertainment (left truncation) rather than true temporal changes in the clinical profile of patients with TC. Because the population became progressively younger during the study period, part of the observed decline in comorbidity prevalence may also be attributable to changes in the age structure of the analyzed cohort.

Finally, the observational design precludes causal inference, and the mechanisms underlying the observed temporal changes in age and the prevalence of recorded comorbidities cannot be directly assessed. Moreover, changes in diagnostic pathways, healthcare utilization, and coding practices over time may have contributed to the observed reduction in the prevalence of recorded comorbidities, independent of true changes in patient health status.

## 6. Conclusions

In this nationwide cohort of 60,604 patients with first thyroid-cancer-associated hospitalization recorded in the database, a broad spectrum of coexisting non-endocrine disorders was identified, demonstrating that the clinical profile of these patients extends well beyond endocrine pathology alone. The progressive decline in endocrine and cardiovascular comorbidities observed between 2012 and 2024 was accompanied by a decrease in the mean age of patients recorded during thyroid-cancer-associated hospitalizations. These findings describe temporal changes observed in the database and may reflect a combination of demographic shifts, evolving diagnostic pathways, healthcare delivery changes, and documentation practices.

Cardiovascular diseases, respiratory disorders, and additional malignancies represented the most prominent categories of accompanying conditions. The coexistence of these disorders suggests that multimorbidity is a common feature among patients hospitalized with thyroid cancer and may influence diagnostic pathways, treatment strategies, and healthcare utilization. Particularly noteworthy was the frequent occurrence of cardiovascular diseases and second primary malignancies, both of which have been recognized as important determinants of long-term outcomes in thyroid cancer survivors. Respiratory complications were also common and may partly reflect advanced disease and metastatic involvement in a subset of patients. These findings support the concept that thyroid cancer should be considered within a broader framework of multimorbidity rather than as an isolated endocrine malignancy. Because detailed cancer-specific characteristics were unavailable, the present study does not allow assessment of the extent to which individual comorbidity patterns are influenced by disease severity or treatment-related factors.

Comprehensive assessment of coexisting conditions and a multidisciplinary approach to care may improve risk stratification, treatment planning, and long-term management of patients with thyroid cancer. Future studies should evaluate the impact of specific comorbidity patterns on treatment intensity, survival outcomes, and competing mortality risks in patients with thyroid cancer.

## Figures and Tables

**Table 1:** Baseline characteristics of patients with thyroid cancer.

Characteristic	Value
Number of patients	60,604
Women	49,794 (82.2%)
Men	10,810 (17.8%)
Mean age	51.1 ± 15.9 years
Median age	51 years
Age range	3–99 years

**Table 2:** Coexisting thyroid disorders among patients with TC.

Disorder	ICD-10	N	%
Hypothyroidism	E03.0	6978	11.5
Non-toxic goiter	E04.0	974	1.6
Thyrotoxicosis	E05.0	196	0.3
Thyroiditis	E06.0	210	0.3
Other thyroid disorders	E07.0	158	0.3
Benign adrenal neoplasm	D350	99	0.16
Hyperparathyroidism	E210	18	0.03
Pituitary disorders	E220	17	0.03
Benign pituitary neoplasm	D352	11	0.02

**Table 3:** Major categories of non-endocrine comorbidities recorded during first hospitalization.

ICD chapter	N	%
Cardiovascular diseases (I)	8256	13.6
Neoplasms other than TC (ICD-10 chapters C and D; malignant, in situ and benign neoplasms)	3059	5.0
Respiratory diseases (J)	1646	2.7
Gastrointestinal diseases (K)	653	1.1
Renal diseases (N)	595	1.0
Musculoskeletal diseases (M)	611	1.0
Infectious and parasitic diseases (A + B)	1 452	2.4%

**Table 4:** Changes in age at diagnosis and prevalence of major comorbidity groups among patients with first thyroid-cancer-associated hospitalization recorded in the database, 2012–2024.

Parameter	Change 2012–2024	Annual Change	*p*
Mean age at diagnosis	53.7 → 49.8	−0.40 years/year	<0.001
Endocrine, nutritional and metabolic disorders (ICD-10 chapter E)	52.0 → 25.3	−1.94 pp/year	<0.001
Cardiovascular diseases (ICD-10 chapter I)	19.9 → 7.7	−0.95 pp/year	<0.001

## Data Availability

The data supporting the findings of this study consist of aggregated administrative data from the Nationwide Hospital Morbidity Study. These data are not publicly available due to legal and privacy restrictions. Access to the underlying data requires permission from the relevant data custodians and compliance with applicable data protection regulations.
